# Ethnobotany, Biological Activities and Phytochemical Compounds of Some Species of the Genus *Eryngium* (Apiaceae), from the Central-Western Region of Mexico

**DOI:** 10.3390/molecules28104094

**Published:** 2023-05-15

**Authors:** Jeanette G. Cárdenas-Valdovinos, Ignacio García-Ruiz, María V. Angoa-Pérez, Hortencia G. Mena-Violante

**Affiliations:** Instituto Politécnico Nacional, Department of Research, CIIDIR IPN Unidad Michoacán, Jiquilpan 59510, Mexico; jcardenasv@ipn.mx (J.G.C.-V.); igarciar@ipn.mx (I.G.-R.); vangoa@ipn.mx (M.V.A.-P.)

**Keywords:** *Eryngium*, Apiaceae, medicinal plants, traditional uses, bioactive compounds, ethanolic extracts, infusion, hypoglycemic activity, hypolipidemic activity

## Abstract

There are approximately 250 species of *Eryngium* L. distributed throughout the world, with North America and South America being centers of diversity on this continent. In the central-western region of Mexico there may be around 28 species of this genus. Some *Eryngium* species are cultivated as leafy vegetables, ornamental, and medicinal plants. In traditional medicine they are used to treat respiratory and gastrointestinal conditions, diabetes, and dyslipidemia, among others. This review addresses the phytochemistry and biological activities, as well as traditional uses, distribution, and characteristics of the eight species of *Eryngium* reported as medicinal in the central-western region of Mexico: *E. cymosum*, *E. longifolium*, *E. fluitans* (or *mexicanum*), *E. beecheyanum*, *E. carlinae*, *E. comosum*, *E. heterophyllum*, and *E. nasturtiifolium*. The extracts of the different *Eryngium* spp. have shown biological activities such as hypoglycemic, hypocholesterolemic, renoprotective, anti-inflammatory, antibacterial, and antioxidant, among others. *E. carlinae* is the most studied species, and phytochemical analyses, performed mainly by high-performance liquid chromatography (HPLC) and gas chromatography coupled with mass spectrometry (GC-MS), have shown its content of terpenoids, fatty acids, organic acids, phenolic acids, flavonoids, sterols, saccharides, polyalcohols, and aromatic and aliphatic aldehydes. According to the results of this review on *Eryngium* spp., they constitute a relevant alternative as a source of bioactive compounds for pharmaceutical, food, and other industries. However, there is a lot of research to be conducted regarding phytochemistry, biological activities, cultivation, and propagation, in those species with few or no reports.

## 1. Introduction

Plants constitute a valuable resource in the health systems of developing countries, and in this sense, the World Health Organization (WHO) has estimated that more than 80% of the world population uses traditional medicine for primary health care, mainly through treatments with plant extracts or their active ingredients [1]. According to the WHO (1979) [2] a medicinal plant is defined as any plant species that contains substances which can be used for therapeutic purposes or whose active ingredients can serve as precursors for the synthesis of new drugs. Therefore, drugs derived from plants have gained relevance both in traditional medicine and in modern medicine [3]. However, Ref. [1] the massive use of medicinal plants is limited by procedures such as the classification, identification and characterization of these and their active principles; therefore, the generation of knowledge about medicinal plants in different latitudes is highly relevant.

In Mexico, medicinal plants have been historically important even before colonization of the New World; thus, knowledge of plants and their usefulness as medicine, have passed from generation to generation as part of inherited traditions. There are people who master the empirical knowledge of medicinal plants, while others only sell them unaware of their properties and health effects [4]. The diversity of plants is very high in Mexico, and between 3000 and 5000 species are plants with therapeutic potential and only 1% have been studied in depth to discover their medicinal properties [5]. Among the plants with medicinal properties are those belonging to the genus *Eryngium*, the largest and most complex of the Apiaceae family. There are 250 species of which 28 are distributed in the central-western region of the country and 21–22 in Michoacán [6]. Therapeutic uses have been reported for some of these *Eryngium* species mostly in rural communities, and some of them have been studied due to their beneficial properties, mainly to treat diabetes, dyslipidemia, and kidney conditions [7,8,9,10]. Extracts from some *Eryngium* spp. have been shown to have biological activities [11,12] and various bioactive compounds [13]. However, only a few species have been investigated to ensure the efficacy and safety of the treatments for which they are prescribed.

Consistent with the above information, the main objectives of the present work were to review (1) the traditional uses associated with the *Eryngium* spp. distributed in the central-western region of the country; (2) the advances in the detection and identification of their bioactive compounds; and (3) the biological activities that reveal their healing potential. Having this information available will allow us to propose future research to contribute to the development of safe products and therapies aimed at treating chronic degenerative diseases, among others.

## 2. Ethnobotany: Description, Distribution, and Traditional Uses

### 2.1. The Genus Eryngium Worldwide and in Mexico

There are approximately 230–250 species of *Eryngium L.* distributed in tropical and temperate regions in Eurasia, North Africa, North and South America, and Australia [14,15]. Except in the tropics and Southern Africa [16,17], *Eryngium L.* is the largest genus and possibly the most taxonomically complex of the Apiaceae family [18]. In his treatise, Wolff (1913) [19] considered *Eryngium* one of the most complex, and he recognized two groups within the genus: “Species Gerontogeae” representing 12 sections of the Old World (Africa, Europe, and Asia) and “Species Americanae and Australienses” including 22 sections from the New World (America and Australia). According to Wörz (1999) [15], and in agreement with updated information, there are about 100 South American and 85 North American species. In Mexico, there are around 50–60 species: between 27 and 28 in the central-western region of the country, and 21–22 in the state of Michoacán [6].

### 2.2. The Genus Eryngium Worldwide and in Mexico

#### *Eryngium* L.

Creeping to erect, herbaceous, caulescent or acaulescent, these plants are usually glabrous biennials or perennials, which grow from stout taproots or rootstocks bearing fibrous roots. The leaves are coriaceous or membranaceous, entire, or pinnately or palmately lobed to divided, often ciliate to spinose, and the venation parallel or reticulate. The petiole’s sheathing is sometimes septate. It has an inflorescence capitate, with the heads either solitary or in cymes or racemes. It has an involucre of one or more series of entire or lobed bracts subtending the head and an involucel of entire or lobed bractlets subtending the flowers. The flowers are white to purple and sessile; the petals ovate to oblong with variously inflexed and lobed to fimbriate tips; the sepals ovate to lanceolate, acute to obtuse, entire, or rarely spinescent; the styles are shorter and do not exceed the sepals, as they lack a stylopodium; and the carpophore is absent. The fruit are globose to obovoid, scarcely flattened laterally, and variously covered with scales or tubercles; the ribs are obsolete; the commissure is broad; and the five oil-tubes are mostly inconspicuous. The seeds subterete in cross-section, and the face is plane or slightly concave [20].

Worldwide, several species of the genus *Eryngium* have been used in traditional medicine, especially to treat cholesterol and diabetes problems. In Mexico there are about 50 species, of which about 27 taxa are considered for the central-west region, which includes the states of Michoacán and Jalisco, as well as areas adjacent to the neighboring states: State of Mexico, Guanajuato, and the southern zone of Nayarit [20, 21,22] (Table 1). Various properties have been reported for only eight taxa since they are traditionally used as an auxiliary or complement to family health.

The species reported in this review fall into the section and subsection at the infrageneric level, according to Wolff (1913) [19] and Calviño et al. (2008) [18], as “American and Australian species (new world)” as follows (Table 2).

Some characteristics on the biology and distribution of the *Eryngium* species reported in the central-western region of Mexico are shown in Table 3 [6].

### 2.3. Traditional Uses

Many *Eryngium* species have traditionally been used as ornamental, edible, or medicinal plants [24]. Various *Eryngium* spp. are used for the treatment of different inflammatory diseases around the world [25,26,27]. In Mexico, the use of different *Eryngium* species in traditional medicine is very important, some of them are distributed in the central-western region (Table 4). In general, they are known as “hierba del sapo” (toad grass) regardless of the species. Such is the case of *Eryngium beecheyanum* Hook. F. and Arn., a species mostly preferred by the population of the Purépecha Plateau in Michoacán, Mexico, to treat skin inflammation; consumed orally as an infusion of the aerial part of the plant, it is also applied in fomentation form to the affected area [28]. It has been reported as an antipyretic [29], and the farmers of the Sierra de Huautla, Morelos, use it to treat renal inflammation [10].

*Eryngium carlinae* F. Delaroche, also known as “hierba del sapo” or “mosquitas” [12,30,31], whose medicinal properties have been widely studied, is also one of the most used in indigenous communities in central Mexico as well as in Michoacán, Hidalgo, State of Mexico and Querétaro [32,33]. It is mainly used in the treatment of kidney problems such as cystitis known as “mal de orín” in Puebla and Tlaxcala, kidney pain in Hidalgo, and as a diuretic in the State of Mexico. In general, the whole plant is used with or without the roots and taken orally as an infusion. It is also used to reduce inflammation of the stomach in the State of Mexico, and to treat biliary disease, taken on an empty stomach. In the case of inflammation caused by blows, it is topically applied to the affected area, using hot fomentations, and adding salt to the infusion. *E. carlinae* is also used to treat inflammatory conditions of the bowel, back pain, pain of the bones, chest, ear, and hernia, and against snake bites and fever [9,32]. One of the most popular uses is in the treatment of diabetes and dyslipidemias as an infusion taken throughout the day called “agua de uso” [9,32]. Additional uses have been reported, including as an aphrodisiac, an anticrotalic antidote, antispasmodic, antipodagric, antitumor, carminative, diuretic, expectorant, increased heat in the stomach, cold-caused illnesses, asystole, and gastroenteritis, among others [29].

*Eryngium comosum* F. Delaroche, is used as an aphrodisiac, antigonorrheal, antipyretic, antipodagric, diuretic and oxytocic [29], for lipid-lowering, and to treat “mal de orín” (cysititis) related to urinary tract infections and kidney pain [34].

*Eryngium longifolium* is another of the species used to treat diabetes in Hidalgo, where it is given the name “piñuela”, where the form of use is as an infusion of the dried plant (aerial part) taken throughout the day (“agua de uso”) [35,36]. In addition, its use as a diuretic, emmenagogue, and alexiteric has been reported [37], similar to *Eryngium fluitans* [38]. Another empirically well-known species is *Eryngium heterophyllum* Engel, also called “toad grass”, which is considered useful for the treatment of diabetes, arthritis, and hypercholesterolemia, among other diseases. The commonly recommended form of use is to prepare the aerial part of the plant in its natural state, pulverized and boiled in water, and the infusion is supplied according to the condition [4,8]. It has also been reported to be useful for the control of gallstones [23].

*Eryngium nasturtiifolium* is known to be used locally as a traditional medicine against type 2 diabetes mellitus and “mal de orín” [39], similar to other *Eryngium* species.

The traditional medicinal uses of *Eryngium* species reviewed are summarized in Table 4.

**Table 4:** *Eryngium* spp. distributed in the central-western region of Mexico with reports of medicinal use.

Scientific Name	Common Name	Medicinal Uses	Preparation	References
*E. beecheyanum*	Hierba del sapo	Antipyretic	Infusion of the whole plant; fomento	[29]
		For kidney inflammation	NR	[10]
		For skin inflammation	Infusion of the aerial part, taken orally; additionally, it is used in the fomentation form on skin	[28]
*E. carlinae*	Hierba del sapo, mosquitas	Aphrodisiac, anticrotalic, antispasmodic, antipodagric, antitumor, asystole, carminative, diuretic, tonic, and expectorant; in cold-caused diseases, it increases heat in the stomach; and to treat gastroenteritis	Infusion of the whole plant	[29]
		To treat type 2 diabetes, dyslipidemias, and digestive problems; and to control blood pressure	Infusion	[8,9]
		Diuretic and antipyretic; to treat kidney problems, “mal de orín” (cystitis), and kidney pain; to control bile (taken on an empty stomach); to treat stomach and intestine inflammation, pain in the back, bones, chest and hernia; for snake bites; and in piercing ears	Infusion, whole plant with or without root, it is taken orally	[33]
		For inflammations due to blows	It is applied topically, through hot fomentations adding salt to the infusion	[33]
*E. comosum*	Hierba del sapo, piñitas	Aphrodisiac, antigonorrheal, antipyretic, antipodagric, diuretic, and oxytocic	Infusion of the whole plant	[29]
		Hypolipidic; to treat cystitis (“mal de orín”)	NR	[34]
*E. cymosum*		To treat type 2 diabetes; and as a hypoglycemic	Infusion: as “agua de uso”	[8,9,40]
*E. fluitans*		Diuretic, emmenagogue, and alexiteric	NR	[38]
*E. heterophyllum*	Hierba del Sapo	To treat diabetes, arthritis, and hypercholesterolemia; and to control bile and reduce gallstones related to emotional problems (e.g., anger)	Infusion, whole plant	[4,41]
		To control of gallstones	NR	[23]
*E. longifolium*	Piñuela	To treat type 2 diabetes; and as a diuretic, emmenagogue, and alexiteric	Infusion aerial part of the plant: as “agua de uso”	[35,36,37,38]
*E. nasturtiifolium*	Hierba del sapo	To treat type 2 diabetes and cystitis (“mal de orín”)	Infusion whole plant: as “agua de uso”	[39]

NR—Not reported.

## 3. Biological and Pharmacological Activity

The use of plants in medicines ranges from crude preparations or extracts, to refined extracts and single molecular species. In terms of categories of use, these encompass food supplements, herbal medicines, botanical drugs, and prescription medicines. There is an increasing interest in plants as a source of novel pharmacophores [42].

In this context, pharmacological studies of medicinal plants have been carried out, addressing various extract evaluation strategies in vitro or in vivo, using different extractive solvents or following traditional preparation practices. The plants of the genus *Eryngium* have not been the exception; thus, the evaluation of the extracts of *Eryngium* spp. distributed around the world, have shown multiple beneficial effects [43], such as anti-inflammatory [44], against snake and scorpion venoms [45,46], antibacterial, antioxidant [47], antihyperglycemic [48], and cytotoxic against human tumor cell lines [49], among others.

Regarding the *Eryngium* spp. reported in the central-western region of Mexico, records of its use in traditional medicine were found for eight species, but reports of biological activities were found only for five of these species.

### 3.1. Eryngium carlinae

There has been great interest in learning about its effects on diabetes control; thus, Noriega-Cisneros et al. (2012) [50] investigated the effect of chronic administration of ethanolic extract of *E. carlinae* on glucose, creatinine, uric acid, total cholesterol, and triglyceride levels in the serum of streptozotocin (STZ)-induced diabetic rats. Treatment with ethanolic extract of *E. carlinae* prevented the increase in glucose, triglycerides, total cholesterol, and uric acid in serum; it also reduced the levels of creatinine, uric acid, total cholesterol, and triglycerides in healthy rats compared to those with diabetes. Additionally, ethanolic extract significantly decreased glycosylated hemoglobin (HbA1c) in the serum of diabetic rats. The authors concluded that administration of *E. carlinae* reduced cardiovascular-risk-related hyperlipidemia in diabetes mellitus. Subsequently, Noriega-Cisneros (2013) [51] analyzed the chemical composition of the ethanolic extract of *E. carlinae* and studied the effect of its consumption in STZ-induced diabetic rats, and its antioxidant activity was assayed. The results showed that ethanolic extract had no hypoglycemic effect when administered orally to diabetic rats (45 mg/kg); however, it did reduce cholesterol and triglyceride levels, improving the lipid profile and reducing the cardiovascular risk index. The in vitro analysis showed antioxidant activity and a considerable amount of flavonoids and phenolic compounds related to it; however, the in vivo analysis did not have a significant effect on lipid peroxidation, and antioxidant enzymatic activity of the superoxide dismutase (SOD) and catalase (CAT) only showed an effect on reducing the nitric oxide levels. Histological analysis of the kidney showed that although the ethanolic extract of *E. carlinae* did not control hyperglycemia, it may offer benefits on lipid profile and progression of renal damage. Later, Noriega-Cisneros et al. (2020) [31] investigated the mechanism of action of the hypolipidemic effect of the ethanolic extract of *E. carlinae*, analyzing its composition and lipid-lowering activity. The extract was administered orally to STZ-induced diabetic rats (30 mg/kg) for more than 40 days, and its effect was compared with that of atorvastatin (a drug used to lower cholesterol levels). The analyzed extract reduced total cholesterol and non-high-density lipoprotein cholesterol (C-HDL) levels and increased the C-HDL levels reduced in diabetes, decreasing the atherogenic index and, therefore, the risk of suffering cardiovascular disease risk at the same level as atorvastatin. The results demonstrated the hypolipidemic potential of ethanolic extract of *E. carlinae* and support its use in traditional medicine as a hypolipidemic agent. On the other hand, García-Cerrillo et al. (2018) [52] demonstrated that the hexanic extract of *E. carlinae* had in vitro and in vivo antioxidant activity associated with the decrease in glucose and triacylglyceride levels during hyperglycemia and suggested that this effect could reduce the risk of developing diabetic cardiomyopathy. The authors administered hexanic extract of *E. carlinae* (30 mg/kg) to STZ-induced diabetic rats for seven weeks and found that serum levels of glucose, triacylglycerides, and TBARS (thiobarbituric acid reactive substances) were significantly reduced in diabetic rats supplemented with the extract. Peña-Montes et al. (2019) [30] also evaluated the in vitro antioxidant activity of the hexanic extract of *E. carlinae* inflorescences in *Saccharomyces cerevisiae* under stress induced by hydrogen peroxide, and later, they tested the extract in STZ-induced diabetic male Wistar rats. The hexanic extract showed in vitro antioxidant activity at different concentrations compared to ascorbic acid (positive control). Oral administration (30 mg/kg) of the hexanic extract reduced blood glucose levels; lipid peroxidation in the liver, kidney, and brain; protein carbonylation; and reactive oxygen species (ROS) production in normoglycemic and hyperglycemic rats. CAT activity in the brain, kidneys, and liver also increased. These findings showed the antioxidant properties of the hexanic extract of *E. carlinae* inflorescences.

Regarding active metabolites, Castro-Torres et al. (2017) [53] determined the hypocholesterolemic activity of the hydroalcoholic extract of aerial parts of *E. carlinae* and demonstrated the presence of hexa-*O*-acetyl-d-mannitol and its acetylated derivatives by gas chromatography coupled with mass spectrometry (GC-MS) analysis. The authors concluded that mannitol promoted osmotic diuresis, which may favor cholesterol transport, preventing it from accumulating in enterocytes and the development of hypercholesterolemia; in this sense, mannitol-based drugs are used to promote diuresis (before irreversible renal failure) and urinary excretion of toxic substances as an antiglaucoma agent, and as an aid in the diagnosis of renal function [54]. In the same way the diuretic effect and the excretion of toxic substances, the renoprotective activity of *E. carlinae* has been reported by Pérez-Ramírez et al. (2016) [55]. The authors studied the effect of plant decoctions on renal dysfunction in high-fructose and high-fat fed rats. Decoction consumption reduced serum uric acid, urine albumin and urea, and increased creatinine clearance, which was associated with reduced hyperglycemia, renal lipid accumulation, and oxidative stress. These results suggested that *E. carlinae* could be used as an ingredient of functional beverages with renoprotective effects.

The only clinical study of *E. carlinae* found during this review was reported by Montes-Moreno (2017) [32]. The authors evaluated the effect of consuming aqueous extracts of toad grass on serum triglycerides, body composition, and anthropometric values in adults. A randomized, parallel blind clinical trial was carried out, and anthropometric measurements, body composition, and blood biochemistry were taken. Individuals with triglycerides >150 mg/dL were selected to determine the effect of drinking the aqueous extract on the baseline parameters of the participants after four weeks. The consumption of the extract reduced weight and body fat (approx. 1 kg) and triglycerides and VLDL cholesterol (21%). The results obtained suggested that drinking the infusion at a 1% concentration at least once a day could reduce and/or control high serum triglyceride levels and be an adjuvant in reducing the percentage of body fat and weight.

Another reported use of *Eryngium* spp. is the treatment of cholelithiasis; therefore, Valdivia-Mares (2021) [56] evaluated the effectiveness of a 50% hydroalcoholic extract of *E. carlinae* to treat cholelithiasis by an in vitro dissolution model using 30 stones formed by ≥70% cholesterol selected from 1597 stones obtained by cholecystectomy. To improve solubility and resemble gallbladder conditions, the test temperature was between 35 and 37 °C, and the extract was renewed every hour for 20 h. Solutions of 50% ethanol and 99% ethyl ether were used as negative and positive controls, respectively. The dissolution rate of the media was estimated as the reduction in the mass of the treated stones (g/mL/h). The extract showed a higher dissolution rate (0.00280–0.00285 g/mL/h) than that shown by ethanol (0.00255 g/mL/h) and six times lower than that shown by ethyl ether (0.00715 g/mL/h). The authors suggested that these results could contribute to the development of a safer, cheaper, and less invasive therapy, such as a product containing *E. carlinae*.

Another of the benefits attributed to *E. carlinae* is its antispasmodic activity, which was confirmed in vivo by Pérez-Gutiérrez et al. (2006) [57]. This activity was attributed to the presence of two γ-lactones from the methanol fraction isolated and characterized by the authors.

Regarding the antimicrobial activity of *E. carlinae*, tests performed in vitro have not shown significant growth inhibition of human pathogenic bacteria [11]. On the other hand, Galindo-Hernández (2018) [58] evaluated the antifungal activity of the acetonic extract of *E. carlinae* against *Candida* spp. strains isolated from pediatric dental patients. The extract did not show strong antimicrobial activity against *C. albicans* (ATCC 90029). In contrast to the above antimicrobial studies, Espino-Garibay (2010) [59] evaluated the antimicrobial effect of *E. carlinae* metabolites, identifying 21 metabolites in ethanolic extracts (leaves, peduncles, and flowers) by GC-MS. Regarding the volatile compounds, germacrene showed antifungal activity against *Colletotrichum lindemuthianum* (49.6%) and Botrytis cinerea (39.1%). The highest antifungal activity against *C. lindemuthianum* (almost 100%) was shown by spathulenol (50 mg/mL) and piperitone oxide (500 mg/mL). While spathulenol, piperitone oxide, and menthol (100 mg/mL) exerted a less inhibitory effect against *B. cinerea* (37.8%), only piperitone oxide (250 mg/mL) had an inhibitory effect against *Fusarioum oxysporum* (28.8%). On the other hand, the antimicrobial activity of *E. carlinae* terpenoids was lower; thus, pulegone and borneol, with a dose of 500 mg/mL, inhibited the oomycete *Phytophthora* cinammomis 32.1 and 30.8%, respectively. Meanwhile, spathulenol (500 mg/mL) and myrcene (250 mg/mL) exerted an inhibitory effect of 18.1 and 15.3%, respectively. The crude extracts showed higher activity against *P. cinnamomi* (34%).

### 3.2. Eryngium comosum

There are few scientific reports on the biological activities of *Eryngium comosum*, Delaroche F.; for example, the work of Ronquillo de Jesús (2013) [60] who determined the antioxidant activity of ethanolic, aqueous, hexanic, and ether of petroleum extracts of *E. comosum* using the DPPH assay. In addition, the extracts cytotoxicity was assayed in vitro in peripheral blood mononuclear cells and in vivo in *Artemia salina*. Ethanolic and aqueous extracts at a concentration of 1000 ppm showed IC50 values of 4.93 µg/mL and 49.52 µg/mL, respectively. None of the extracts showed toxicity in mononuclear cells, while the extract with petroleum ether did show a cytotoxic effect in *A. salina* (IC50 2.92 ppm).

The antimicrobial activity has also been studied in *E. comosum*, in addition to the antioxidant activity. Díaz-Alvarado et al. (2020) [61] evaluated the antibacterial activity by the disk diffusion method (DDM), using reference strains of equine pathogenic bacteria: *Listeria monocytogenes* ATCC 19115, *Staphylococcus* sp., *Escherichia coli* ATCC 25922, and *Salmonella enterica* serotype *Enteritidis* ATCC 13076. Ethanolic extract of *E. comosum* (50%) prepared with dried tissue (125 mg/mL) inhibited the growth of *Staphylococcus* sp., *S. enterica*, and *L. monocytogenes*, showing a greater effect on the latter strain. The results suggested the extract of *E. comosum* as a source of antimicrobial agents to treat equine infections, although further in vitro and in vivo research is required to achieve its application. In the same way, Díaz-Alvarado (2020) [62] analyzed the bioactive compounds in aqueous and ethanolic extracts (50 and 70%) of *E. comosum*, and assayed antioxidant capacity and antimicrobial activity in 50% ethanolic extracts of this medicinal plant. The 50% ethanolic extract of *E. comosum* showed antioxidant capacity (1973.42 μM ETCA/g) and antibacterial activity against *Enterococcus* sp. and *Salmonella* sp. (inhibition zone diameter = 11.3 mm).

Regarding in vivo studies, Pérez-Reyes (2016) [13] reported that the aqueous extract of *E. comosum* reduced cholesterol and triglyceride levels in rats with dyslipidemia, induced with a hypercholesterolemic and hypertriglyceridemic diet. The extract was administered intragastrically for 3 weeks, testing three doses: 100, 200, and 400 mg/kg. After the treatment, the influence of the aqueous extract on the weight of adipose and muscle tissues was observed; however, a body weight reduction was not reported. On the other hand, the decrease in serum cholesterol levels was recorded at a dose of 100 mg/kg, with a serum concentration of 189.45 mg/dL in the control group and 99.16 mg/dL in the treated group; while the serum concentration of triglycerides decreased only with the 200 mg/kg dose, being 403.1 mg/dL in the control group and 337.8 mg/dL in the treated group.

### 3.3. Eryngium cymosum

One of the most widespread traditional uses of *Eryngium* ssp. is the treatment of type 2 diabetes (T2D), and there are some reports about its hypoglycemic effect. For example, the study carried out by Espinoza-Hernández et al. (2021) [8], in which the aqueous extract of aerial parts of the *E. cymosum* plant was administered via gavage to Wistar rats with streptozotocin-nicotinamide-induced hypoglycemia (STZ-NA). The authors reported the antihyperglycemic effect of the extract in the pyruvate tolerance test and the significant reduction of postprandial hyperglycemia in the maltose tolerance tests. As the main mechanism of action, the extract suppressed gluconeogenesis by inhibition (almost 100%) of the enzymes glucose-6-phosphatase (G6Pase) and fructose-1,6-bisphosphatase (FBPase), which is the altered pathway that causes fasting and postprandial hyperglycemia in patients with T2D; the extract also reduced the activity of a-glucosidases by 32%. In addition, it decreased insulin levels when it was administered orally in healthy rats in both nutritional states, without affecting normoglycemia in normal curves and reducing the postprandial peak in glucose load curves. The authors concluded that the traditional form of consumption of *E. cymosum* is safe and regulates glucose levels both fasting and in the postprandial state.

Subsequently, the same research group published a study that evaluated the chronic effects of traditional extracts on hyperglycemia and hypertriglyceridemia of some Mexican medicinal plants, including *E. cymosum* [9]. The aqueous extract was administered via gavage to hyperglycemic STZ-NA Wistar rats, daily for 42 days. For the preparation of the extract, 20 g of dried and ground plant material (aerial parts) were added to 500 mL of boiling, distilled water for 15 min. Non-fasting blood glucose (NFBG), HbA1c, and blood triglycerides were determined. The authors confirmed the long-term efficacy of the extract, as *E. cymosum* prevented the worsening of hyperglycemia by avoiding the significant increase in glucose levels shown by the negative control group and the increase in HbA1c (2.98%). Despite its antihyperglycemic effects, the extract was less effective in controlling triglycerides. The authors generated evidence of the antihyperglycemic effect of this Mexican medicinal plant, as well as its long-term efficacy in the control of T2D.

Research to reveal the mechanisms of action of the hypoglycemic effect of *E. cymosum* has led to the description of a new metabolite, acylated flavonol, and the isolation of known compounds both in aqueous extract and butanolic extract [40], whose chemical structures were elucidated using spectroscopic techniques, as described in the following section. Additionally, the role of the acylated flavonol glucoside on the inhibition of G6Pase and FBPase has been demonstrated.

### 3.4. Eryngium heterophyllum

Some studies have been carried out in *E. heterophyllum* to confirm its anti-inflammatory, hypoglycemic, and hypocholesterolemic activity. Navarrete et al. (1990) [63] reported the decrease in rat serum cholesterol when the aqueous extract of *E. heterophyllum* was administered orally. For his part, Miranda-Velásquez (2010) [64] tested the hypocholesterolemic activity of crude extracts dissolved in water or in a Tween 80/saline solution at two doses of 50 and 100 mg/kg of weight administered to hypercholesterolemic mice for five days, which at the end of this period were fasted for 12 h. The results showed that only the aqueous extracts of *E. heterophyllum* at 100 mg/kg showed a cholesterol reduction (20.7%). Therefore, this extract was subsequently evaluated in vitro using Vero cells to determine the inhibitory effect of the HMG-CoA enzyme. The results showed that indeed the mechanism of serum cholesterol reduction was related to the inhibition of said enzyme, as in the case of statin drugs. In the same way, García-Gómez et al. (2019) [65], in a 1-month clinical study, showed that combined treatment of *E. heterophyllum* and *Amphipterygium adstringens* with proven hypocholesterolemic activity tested in rats, reduced triglyceride levels by an average of 20%. On the other hand, Carreón-Sánchez et al. (2013) [4] showed that the ethanolic extract of *E. heterophyllum*, after being administered to mice by oral gavage in a single dose of 100 mg/kg of weight in a volume of 0.2 mL/30 g, had no hypoglycemic effect or acute or chronic anti-inflammatory effect; nor did it cause visible toxic effects in the acute poisoning model in mice.

Molina-Garza et al. (2014) [66] conducted a study to screen the trypanocidal activity of the *Eryngium heterophyllum* plants used in traditional Mexican medicine for the treatment of various diseases related to parasitic infections. Cultured *Trypanosoma cruzi* epimastigotes were incubated for 96 h with different concentrations of methanolic extract of *E. heterophyllum*, and the inhibitory concentration (IC50) was determined. The methanolic extracts exhibited the highest trypanocidal activity (88–100%) at a concentration of 150 µg/mL.

### 3.5. Eryngium longifolium

A dose-independent hypoglycemic effect has been reported for *E. longifolium*. Andrade-Cetto et al. (2021) [35] evaluated aqueous (30 and 310 mg/kg doses) and ethanolic (32 and 318 mg/kg doses) extracts of the aerial parts of the plant in hyperglycemic STZ-NA Wistar rats. Previously, the authors determined the basic phytochemical profiles (see next section) and acute toxicity tests, which did not show any physical problems or behavioral changes after oral administration of the maximum dose of 2000 mg/kg body weight (b.w.) of each extract; no deaths were reported and the LD50 was higher than the maximum dose used. In addition, they tested the inhibition of the G6Pase and FBPase enzymes involved in glucose metabolism. This study validated for the first time the traditional use of the aerial part of *E. longifolium* as a hypoglycemic agent in a hyperglycemic animal model; the results indicated that the in vitro inhibition of G6Pase and FBPase could be associated with the hypoglycemic effect in vivo. Therefore, the authors concluded that the ability to regulate hyperglycemia could involve inhibition of hepatic glucose production, which primarily controls fasting glucose levels, and that the doses traditionally consumed did not generate toxic effects.

According to the previous information, the main potential of the Mexican species of *Eryngium* to promote health, is related to lipid metabolism, which has been proven by the capacity of its extracts to decrease cholesterol, triglycerides, and body fat levels; this has also been reported for other species of *Eryngium* [67].

It is important to highlight the potential of the aqueous extracts of *E. cymosum* and *E. longifolium* for the control of diabetes, as reported for *E. foetidum* and *E. billardieri* [67,68]. The information found about hypoglycemic activity shows the differences between the biological and pharmacological activity that different Mexican species of *Eryngium* show; in addition, such differences could be associated with the type of extract evaluated since the content and nature of the active ingredients will also vary. For instance, the acetonic and methanolic extracts of *E. foetidum* did not show antibacterial activity against *Escherichia coli*, *Salmonella infantis*, *Listeria monocytogenes*, *Staphylococcus aureus*, or *Bacillus cereus* [69], while the essential oil of *E. maritimum* showed a significant antibacterial activity against *L. monocytogenes* and *E.coli* due to its content of oxygenated sesquiterpenes [70], and the leaf hydromethanolic extract of *E. maritimun* showed antimicrobial activity against *S. aureus*, *B. cereus*, *Salmonella enterica*, *Pseudomonas aeruginosa*, *P. fluorescens*, *P. marginalis*, *E. coli*, and *Erwinia carotovora* subsp. carotovora [71]. Although many species of *Eryngium* have shown antimicrobial activity against Gram-positive and Gram-negative bacteria, some species of fungi and yeasts, and viruses, it has been suggested that multi-target antimicrobial experiments should be carried out using extracts of *Eymgium* spp. as antimicrobial agents in order to expand the knowledge about its antimicrobial potential [72].

Additionally, it is important to point out that the study of the anticholelithiasis and trypanocidal activity shown by *E. carlinae* and *E. heterophyllum*, respectively, could extend to other species of *Eryngium*; likewise, other activities such as anticlastogenic, anticarcinogenic, antihelmintic, and larvicidal, amongst others reported for *E. foetidum* could be evaluated [43].

The biological activities reported for *E. carlinae*, *E. comosum*, *E. cymosum*, *E. heterophyllum*, and *E. longifolium* are summarized in Table 5.

## 4. Phytochemistry

### 4.1. Screening, Detection, and Identification of Metabolites

Phytochemical characterization is valuable to reveal the presence and identity of secondary metabolites in a plant as well as a helpful tool in the search for bioactive compounds useful for the synthesis of new drugs and other products. The presence of various groups of metabolites has been described in plant extracts (ethanolic, aqueous, methanolic, and hexane) of aerial parts (stems, leaves, and inflorescences) qualitatively analyzed to determine the presence of flavonoids, tannins, terpenoids, and saponins. Additionally, more precise techniques such as GC-MS, nuclear magnetic resonance (NMR), and high-performance liquid chromatography (HPLC) are performed to detect, quantify, and identify metabolites in plant extracts. Regarding the genus *Eryngium*, investigations have been carried out on the phytochemical profile of some *Eryngium* spp. found in the central-western region of México, mainly *E. carlinae*, *E. comosum*, *E. cymosum*, and *E. longifolium*.

The most widely used methods to determine the general phytochemical composition of a plant are screening and the colorimetric techniques of ultraviolet-visible spectrophotometry (UV-Vis). Phytochemical screening involves a series of chemical reactions that make it possible to qualitatively detect the groups of secondary metabolites present in plant extracts [73,74,75,76,77,78]. For example, *E. carlinae*, one of the most common species used along the region delimited for this review, was qualitatively analyzed by Knauth et al. (2018) [11] to determine the main groups of metabolites in methanolic extract from the aerial part of the plant. The authors reported a strong presence of tannins and saponins, as well as a slight presence of flavonoids. Galindo-Hernández (2018) [58] evaluated the phytochemical profile of *E. carlinae*, confirming the presence of triterpenoids, sterols, tannins, coumarins, carboxyls, flavonoids, and carbohydrates. In the same way, Pérez-Reyes (2016) [13] performed phytochemical screening of an aqueous extract of *E. comosum*, reporting the presence of alkaloids, flavonoids (flavones and xanthones), triterpenoid saponins, reducing sugars, tannins derived from catechol, phenolic compounds, and benzoquinones; in addition, the author reported in vivo hypolipidemic activity.

Using UV-Vis, it is possible to determine compound concentration in a solution; this is a simple, reliable and low-cost analytical technique [79]. In this sense, Lemus-de la Cruz-Hurtado et al. (2023) [12] determined and quantified total phenolic compounds (TPC), total flavonoids (TF), and total terpenoids (TT) in aqueous extracts of aerial parts of *E. carlinae*. The authors reported concentrations of 0.0038 mg gallic acid equivalents per mL (mg GAE/mL) for TPC, 3.3032 mg quercetin equivalents per mL (mg QE/mL) for TF, and 0.0424 mg linalool equivalents per mL (mg LE/mL) for TT. Montes-Moreno (2017) [32] reported higher TPC values in aqueous infusions and decoctions of the same species. The authors evaluated by UV-Vis, up to 2.5 mg GAE/mL of TPC and, in the case of TF, the author reported less than 0.2 mg QE/mL. Similarly, Díaz-Alvarado et al. (2020) [61] reported the total phenolic content (4.33 mg GAE/g dry weight) and total saponins (62.2 mg/g dry weight) of the ethanolic extract of aerial parts of *E. comosum*. In the same sense, Díaz-Alvarado (2020) [62] determined the phytochemical profile of ethanolic extracts (50 and 70%) of aerial parts of *E. comosum* by UV-Vis, confirming the presence and abundance of phenolic compounds (up to 4.3 mg GAE/mL), TPC (up to 13.33 mg/g DW), and total saponins (up to 29.33 mg/g DW), with 70% ethanol being the best extractor.

Within the phenols group, phenolic acids (caffeic, rosmarinic, and chlorogenic) and their derivatives are the most abundant in the genus *Eryngium*; these compounds have been analyzed and identified by more precise techniques, such as chromatography [80]. Andrade-Cetto et al. (2021) [35] described the chromatographic profile of the ethanolic extract of aerial parts of *E. longifolium* analyzed by HPLC, the authors detected three signals at wavelengths of 254 and 320 nm with retention time (RT) between 6 and 13 min, which were identified as caffeic, chlorogenic, and rosmarinic acids by comparing them to their commercial standards, rosmarinic acid being the most abundant in the analyzed extract. Likewise, the authors reported other minor intensity peaks with RT between 16 and 24 min, which were attributed to isoflavone-type compounds and glycosylated flavonoids. In a similar work, Espinoza-Hernández et al. (2021) [8] analyzed aqueous extracts of *E. cymosum* by HPLC, finding caffeic, chlorogenic, and rosmarinic acids between 6 and 13 min of RT in the same UV-spectrum (320 nm). Additionally, the chromatographic profile of fractions isolated from aqueous, methanolic, and organic extracts of the same species has been analyzed by Romo-Pérez et al. (2022) [40], confirming that chlorogenic and rosmarinic phenolic acids were the most abundant in aqueous and methanolic extracts, while caffeic and protocatechuic acid were more abundant in the butanolic organic extract; in which, the authors reported for the first time the presence of the flavonoid kaempferol-3-*O*-(2,6-di-*O*-trans-ρ-coumaryl)-β-d-glucopyranoside in *E. cymosum*, associated directly with the hypoglycemic activity shown by the extracts. The identity and structure of the above compounds were corroborated by NMR.

Montes-Moreno (2017) [32] analyzed, by HPLC with a diode array detector coupled to mass spectrometry (HPLC-DAD-MS, detection wavelengths: 280, 320, and 370 nm) aqueous extract obtained by infusion and decoction at different concentrations (1 and 2%) and cooking times (5 and 10 min) of aerial parts of *E. carlinae*. The author reported the presence of hydroxybenzoic acids, mainly gallic acid (1300–2600 µg·mL^−1^, RT = 12.5 min), and to a lesser extent 4-hydroxybenzoic and protocatechuic acids. Likewise, the next hydroxycinnamic acids were found: chlorogenic acid (400–700 µg·mL^−1^, RT = 2.1 min), rosmarinic acid (40–58 µg·mL^−1^, RT = 25 min), and caffeic acid (12–21 µg·mL^−1^, RT = 16.3 min), while ellagic, p-coumaric, ferulic, and sinapic acids were present in the extract in smaller amounts; flavanols such as gallocatechin gallate (600–1600 µg·mL^−1^, RT = 20.4 min), catechin, epicatechin, and epigallocatechin gallate; and flavonols such as quercetin (89–179 µg·mL^−1^, RT = 25.5 min), rutin, and kaempferol were found in the extract as well. Additionally, the presence of flavanones and hydroxybenzaldehydes such as eriocitrin, naringenin, hesperidin, and vanillin were detected in minimal amounts. Using the same method, the author detected β-sitosterol, stigmastanol, sitosteryl-3-β-glucoside, and campesteryl-3-β-glucoside, phytosterols that could help control cholesterol levels in diseases such as hyperlipidemia, hypercholesterolemia, and atherosclerosis [81]. Likewise, the presence of glycosylated saponins involved in anti-inflammatory and antioxidant activities such as α-l-arabinopyranoside phytolaccagenic acid and β-d-glucopyranoside hederagenin was confirmed [82].

Pérez-Ramírez et al. (2016) [55] evaluated the phytochemical composition of the E. carlinae decoction by HPLC with a diode array detector (HPLC-DAD), as well as its participation in the modulating activity of renal dysfunction. Ellagic acid was the most abundant phenolic compound (38.3 areas under the curve, mA), followed by caffeic (20.3 mA), protocatechuic (11.9 mA), and p-hydroxybenzoic (9.8 mA) acids; likewise, flavonoids such as rutin (14.1 mA), catechin (12.1 mA), and epicatechin (11.7 mA) were detected and quantified. Regarding the presence of sterols, the phytosterol α-7-stigmasterol (18.7 mA) was detected as the most abundant, followed by β-sitosterol (11.1 mA), β-campesterol (8.7 mA), and stigmastanol (8.4 mA). Finally, the authors also detected and quantified two major saponins, campesteryl β-d-glucopyranoside (28.9 mA) and sitosteril β-d-glucopyranoside (20.1 mA).

The GC-MS technique is also a useful tool that allows the identification of volatile or semi-volatile compounds present in plant tissues. In this sense, mainly aromatic aldehydes, sesquiterpenes, and fatty acids have been detected in extracts of roots, stems, leaves, and inflorescences of different *Eryngium* spp. using GC-MS. Components such as 2-dodecenal, 2,4,6-trimethyl-benzaldehyde, d-elemene, a-bisabolol, α, and β-selinene, and fatty acids such as palmitic and stearic acid are common in a wide variety of *Eryngium* spp. found around the world, and their direct and synergistic participation in biological activities such as antiprotozoal [83], antibacterial [47,70,84,85], antifungal [47], antioxidant [84,86], and antidiabetic have been reported [86].

In relation to the species found in Mexico, Peña-Montes et al. (2016) [30] reported the presence and abundance of terpenes and sesquiterpenes identified in hexanic extracts of *E. carlinae* inflorescences. The major constituents identified were (Z)β-farnesene (38.79%), β-pinene (17.53%), calamenene (13.30%), and α-farnesene (10.38%). The authors attributed the ability to reduce lipid peroxidation and protein carbonylation to farnesene and pinene, in addition to the probable synergistic effect with other compounds present in the analyzed extract.

Likewise, Espino Garibay (2010) [59] found 21 terpenoid compounds in the ethanolic extract of leaves and inflorescences of *E. carlinae*. The main terpenoids that the author reported were borneol (367 mg/L), α-pinene (278 mg/L), myrcene (256 mg/L), caryophyllene (225 mg/L), and β-pinene (120 mg/L). In addition, the author reported differences in the presence and abundance through different phenological stages of the plant (before, during, and after flowering and fruiting), with the extracts of leaves and inflorescences during and after flowering being those that presented a higher content of metabolites; the isomers of pinene and farnesene were constant in both mentioned stages.

Noriega-Cisneros et al. (2019) [31] reported sesquiterpenes α-selinene (17.54%) and β-selinene (26.04%) as main components of ethanolic extract of aerial parts of *E. carlinae*, in addition to palmitic (14.43%) and stearic acid (14.53%), and others in proportions of less than 5%, such as humulene, stigmasterol, elemol, elemene, and α-cedrene; the authors conclude that these compounds, individually or synergistically, could be involved in the hypolipidemic and hypoglycemic response demonstrated in in vivo studies.

Likewise, some oligosaccharides and polyalcohols have been detected in extracts of leaves and stems of *E. carlinae*. Arana-Argáez et al. (2021) [87] described the presence of D-(−)-fructofuranose, D-(−)-fructopyranose, D-(−)-tagatofuranose, and sucrose, in addition to the polyalcohol 1,5-anhydro-d-sorbitol and cinnamic acid; the authors attributed the anti-inflammatory response, shown in vivo by the extract, to cinnamic acid.

The above information is summarized in Table 6.

According to the literature reviewed, the most studied species is *E. carlinae*, one of the most common in the central-western region of Mexico. However, research is needed on the phytochemical composition of the other species described in the region. In this sense, in the working group, a preliminary analysis of the phytochemical profile of several *Eryngium* species found in Michoacán and Jalisco, including *E. carlinae*, *E. heterophyllum*, *E. nasturtiifolium*, and *E. beecheyanum*, has been performed using HPTLC, and it has been possible to determine the presence of chlorogenic, rosmarinic, and caffeic acids in ethanolic extracts of inflorescences and stems.

An important aspect to consider when studying or proposing the application of *Eryngium* spp. bioactive compounds, is the extraction methods. *Eryngium* extracts contain various metabolites (e.g., tannins, phenolic acids, saponins, and terpenoids) associated with biological activities that support their use in traditional medicine or reveal new therapeutic potential, serving as targets for developing novel drugs. In order to extract bioactive compounds from plants, different methods are used [108]. Thus, multiple solvents chosen based on the polarity have been used to extract phytochemicals. While the ultrasound-assisted extraction (greater than 20 kHz) is used to disrupt plant cell walls, which helps improve the solvent’s ability to penetrate the cells and obtain a higher extraction yield, the use of a low operating temperature through processing allows the procurement of a high extract quality reducing the amount of solvent and energy used [109,110]. Unfortunately, there are still some concerns related to experimental repeatability and reproducibility [108]. Microwave-assisted extraction (MAE) has been used as an alternative to conventional techniques for the extraction of biocompounds due to its important advantages, among which are controllable and effective heating, faster energy transfer, the reduction of extraction time and use of solvents, higher selectivity, and enhanced yield [111,112,113]. Furthermore, new nonconventional technologies are emerging offering superior extraction efficiency in terms of cost, yield, extraction time, and/or selectivity [114]. In this sense, green synthesis of nanoparticles using plant extracts is a promising alternative to chemical methods [115,116,117].

### 4.2. Properties of Phytochemical Compounds in Species of the Genus Eryngium

Rosmarinic, caffeic, and chlorogenic acids are amongst the phenolic compounds reported in the genus *Eryngium*, regardless of the studied species. Rosmarinic acid (RA, molecular formula C_18_H_16_O_8_) named (R)-α-[[3-(3,4-dihydroxyphenyl)-1-oxo-2E-propenyl]oxy]-3,4-dihydroxy-enzenepropanoic acid is an ester of caffeic acid and (R)-(+)-3-(3,4-dihydroxyphenyl) lactic acid and originated from the amino acids L-phenylalanine and L-tyrosine, respectively (Hitl et al., 2020) [118]. RA has been proven to act as (1) an anticarcinogenic, as it inhibits the gene expression, the growth, and the proliferation of tumor cells [119]; (2) an antidiabetic, as it prevents hyperglycemia by increasing the insulin sensitivity index and reducing the levels of blood glucose [120]; (3) an antimicrobial, as it inhibits the growth of Methicillin-resistant Staphylococcus aureus (MRSA), *E. coli*, *Pseudomonas* spp., and *L. monocytogenes*, amongst others, and in addition to the formation of biofilm, it kills planktonic cells [121]; and (4) an antioxidant, as it inhibits the formation of free radicals, the generation of ROS, and lipid peroxidation [120]. In addition, RA has shown hepatoprotective, cardioprotective, antiallergic, antidepressant, anti-aging, nephroprotective, and anti-inflammatory activity [118,122]. Budzianowski et al. (2023) [93] investigated the cytotoxic effect of RA obtained from 50% ethanolic extracts of seedlings of different *Eryngium* species. The authors concluded that the rosmarinic acid-4′-*O*-β-glucopyranoside fraction did not show cytotoxic effects on cell lines involved in cancer development (MCF-7, MDA-MB-231, MCF-12A, HT-29, Caco-2, and OVCAR-3) with an IC_50_ average of 400–700 µM in 24–72 h. RA is used as a bioactive ingredient in supplements, and in its isolated or semipurified form, has not shown toxicity in humans. Jia et al. (2010) concluded that the administration of the RA isolated fraction from depsides salts of *Salvia miltiorrhiza* in only one dose (100–200 mg/kg), did not show toxic effects in humans [123].

Caffeic acid (CA, empirical formula C_9_H_8_O_4_, 3,4-dihydroxycinnamic acid) is a phenolic compound abundant in medicinal plants, with multiple biological activities, such as antioxidant, anti-inflammatory, anticarcinogenic, and neuroprotective [124]. Alam et al. (2022) [125] suggested that CA can intervene in the reduction of oxidative stress by blocking or preventing the formation of ROS molecules in the organism, having a potential antioxidant and anti-aging effect. The authors also determined that the CA could inhibit the formation and migration of tumor cells, decreasing the probability of metastasis in different types of cancer, suggesting that CA alone or in combination with other chemotherapeutic agents/drugs might be suggested to treat and manage cancer.

The chlorogenic acid (CGA) is directly involved in the mitochondrial function, the reduction and prevention of oxidative stress, apoptosis, inflammation, obesity, and diabetes. Hernandes et al. (2020) [124] investigated the cytotoxicity and genotoxicity of CA and CGA on human leukemic cell lines and determined that they showed neither cytotoxicity nor genotoxicity over the analyzed cells. However, CGA induces specific hypomethylation on Jukart cells, which can be beneficial against hematologic malignancies, since ADN altered methylation plays an important role in the pathogenic process. RA, CA, and CGA have been reported in different species of *Eryngium*, such as *E. campestre*, *E. maritimum*, *E. plannum*, *E. creticum*, and *E. alpinum* [75,76,77,91,126], and in some regional species addressed in this review, such as *E. carlinae*, *E. longifolium*, and *E. cymosum*.

Kaempferol (KAE, general empirical formula C_15_H_10_O_6_, 3,4′,5,7-Tetrahydroxyflavone) is part of the frequent appearance of flavonoids in different species of *Eryngium*. KAE is synthesized by condensation of 4-coumaroyl-CoA (C6-C3) with three molecules of malonyl-CoA (C6). KAE and its multiple glycosylated forms have been reported as agents with antitumor, anti-inflammatory, and antioxidant activity. Wang et al. (2018) [127] analyzed the antiproliferative activity of hepatic tumor cells of KAE and its glycosides (Kae-3-*O*-rha, Kae-7-*O*-glu and Kae-3-*O*-rut); the authors showed that KAE in its pure form has antiproliferative concentration-dependent activity (0–100 μM), whereas the glycosylated forms did not show such activity. Likewise, the authors reported that KAE has higher antioxidant and anti-inflammatory activity than its glycosylated forms. The safe use of KAE is being discussed since it could have antimutagenic and genotoxic activity; it also has prooxidant action since at high concentrations it can produce ROS [128]. Additionally, in order to overcome its poor bioavailability and improve its pharmacokinetics, the use of kaempferol nanosuspensions, solid dispersions, and complexes of polysaccharides and oligosaccharides has been developed [129].

The ellagic acid (EA, empirical formula C_14_H_6_O_8_, 4, 4′, 5, 5′, 6, 6′-hexahydrodiphenic acid 2, 6, 2′, 6′-dilactone), a flavonoid compound, has shown activity as a regulator of proinflammatory mediators and it also normalizes the lipid metabolism, in addition to having neuroprotective activity by starting several cell signaling pathways, preventing mitochondrial disfunction, and eliminating free radicals [130]. Likewise, catechin (CAT, empirical formula C_22_H_18_O_10_, catechin 5-*O*-gallate) and its derivatives, ((−)-CAT), ECAT ((−)-epicatechin), ECG ((−)-epicatechingallate), EGC ((−)-epigallocatechin), and EGCG ((−)-epigallocatechin gallate), have antioxidant and anti-aging activity, eliminating free radicals and delaying the extracellular matrix degradation induced by ultraviolet radiation (UV) and skin contamination, activating the collagen synthesis and promoting the inhibition of matrix metalloproteinase [131]. Beyond food and biomedical applications, EA and ellagitannins’ (ET) scientific relevance can be linked to advanced materials such as copolymers, chelating reagents, ion-exchange resins, and materials for electrochemical devices, among others [132].

On its part, the rutin and its derivatives (Rut, C_27_H_30_O_16_, quercetin 3-rutinoside) are flavonoids that have antioxidant, antimicrobial, anticarcinogenic, antidiabetic, antiallergic, antidepressant, antihypertensive, and other effects [133]. In the genus *Eryngium*, the flavonoids KAE, EA, CAT, and Rut have been reported in *E. longifolium* and *E. carlinae.* Quercetin and Rut could be applied in nutritional supplements and innovative complexes and formulations for pharmaceuticals [129].

Among the terpenic compounds in the *Eryngium* species of the central-western region of Mexico (described in *E. carlinae*, Table 6), are the borneol and α- and β-pinene, which are present in the essential oils of several aromatic plants and show activity on blood pressure regulation [134] in addition to showing cytogenetic, gastroprotective, anxiolytic, cytoprotective, anticonvulsant, and neuroprotective effects as well as their effects against H_2_O_2_-stimulated oxidative stress, pancreatitis, stress-stimulated hyperthermia, and pulpal pain [135]. Regarding caryophyllene (β-Caryophyllene (BCP)) and farnesene, they are natural sesquiterpenes that have several biological activities such as antimicrobial, anticarcinogenic, anti-inflammatory, antioxidant, anxiolytic-like, and local anesthetic effects. However, its volatility and poor water solubility limit its application in the pharmaceutical field [136].

### 4.3. Tocixity of Phytochemical Compounds in Species of the Genus Eryngium

Some findings about the toxicity of bioactive compounds detected in the *Eryngium* species reviewed in this work are described below. Knauth et al. (2018) [11] studied the cytotoxicity of extracts from various medicinal plants including *E. carlinae* using the Caco-2 enterocyte cell line. Most of the methanolic extracts from the tested plants showed low cytotoxicity.

Agiorgiti et al. (2018) [137] analyzed the cytotoxic and antitumor activities of five organotin complexes (1–5) with o-hydroxybenzoic or p-hydroxybenzoic acids in vitro and in vivo (in Wistar rats). All complexes exhibited strong cytotoxic activity against all cancer cell lines tested, so they could be used as potential chemotherapeutic agents. However, there is controversy about the safety of p-hydroxybenzoic acid in human health, as there is no evidence of its toxicity in humans [138]. In this sense, Downs et al. (2023) [139] evaluated the relation between accumulation of p-hydroxybenzoic acid and methyl, ethyl, propyl, and butyl esters and the incidence of both malignant and benign breast tumors in patients. The authors concluded that propyl and butyl paraben concentrations are higher on metastatic tissue compared to non-metastatic breast cancer tissue. Likewise, the authors suggested that factors that determine the formation of tumors in breast cancer such as age, menopause status, and family history of cancer could be controlled by changing to a paraben-free lifestyle, especially those present in cosmetics as preservatives, although more extensive studies must be carried out to confirm this association.

Budzianowski et al. (2023) [93] reported a similar effect for AR. The authors evaluated the effect of rosmarinic acid 4′-*O*-β-glucopyranoside (RAG4′) on five human carcinoma cell lines and one non-tumorigenic mammary epithelial cell line (MCF-12A). The highest cytotoxic activity of RAG4′ was observed in the Caco-2 cell line and RAG4′ did not show any effect on the non-tumorigenic cell line MCF-12A, indicating that it might be safe as a cosmetic and pharmaceutical ingredient.

D-mannitol found in *E. carlinae* was evaluated in a model of hypercholesterolemia in mice, and a chronic toxicity test was performed. The mice did not show evident signs of toxicity (e.g., lack of motor coordination, piloerection, or pupil dilation) after receiving doses of 100 and 500 mg/kg of the extract for 4 weeks [53].

Hameed et al. (2016) [140] evaluated the cytotoxic profiles of sinapic acid (SA) belonging to the phenylpropanoid family also present in *Eryngium* species. SA is assumed to be therapeutically beneficial and generally non-toxic. The authors tested a wide range of concentrations in Chinese hamster lung fibroblasts (V79) and human cervical carcinoma (HeLa) cells. Concentrations up to 500 μM and 2000 μM had no effect on the viability of V79 and HeLa cells, respectively, demonstrating that SA had no cytotoxic effects in two different cell lines except at very high concentrations. The dichotomous effect of SA on concentration-dependent cell viability has also been reported: at low SA concentrations cell viability was enhanced, while at high SA concentrations cell proliferation was almost completely inhibited [141,142].

Little is known about the genotoxic/antigenotoxic effects of SA, although there are several studies on the effects of different phenolic compounds [140,142]. The genotoxic effect and clastogenic potential of caffeic, cinnamic, and ferulic acids was examined by Maistro et al. (2011) [143]. The authors used in vitro comet and micronucleus (MN) assays in rat hepatoma tissue (HTC) cells at three different concentrations, for 24 h. No genotoxicity was observed by the comet assay, but clastogenic effects were found in HTC by the MN assay.

The subchronic toxicity of ellagic acid, also present in *Eryngium* species, was analyzed by Tasaki et al. (2008) [144] in F344 rats at five doses in a powdered basal diet for 90 days. No mortality or clinical signs related to the treatments were observed during the entire experimental period. The no-observed effect level (NOEL) was estimated to be 5% (3011 mg/kg b.w./day) for males and the no-observed-adverse-effect level (NOAEL) and NOEL in females were estimated to be 5% (3254 mg/kg b.w./day) and <1.25% (778 mg/kg b.w./day), respectively.

Marcarini et al. (2011) [145] studied the cytotoxic, apoptosis-inducing, genotoxic, and protective effects of rutin in HTC cells and demonstrated that at low concentrations (810 μM) the flavonoid decreased the viability and proliferation of cells after 72 h of treatment; however, at 24 h the authors observed induction of DNA damage, with genotoxic effects but without inducing apoptosis. The authors also observed a protective DNA effect by reducing damage induced by procarcinogenic agents such as benzo(a)pyrene suggesting an important biological activity for this compound, which can contribute to human health through diet. A subsequent report noted that rutin is considered a non-toxic and selective modulator of hypercholesterolemia [146].

It has been reported that the administration of β-sitosterol (BS) in rats does not cause genotoxicity or cytotoxicity [147]. A high level of BS concentrations in blood has been correlated with increased severity of heart disease in men who have previously suffered heart attacks [148]. An extensive toxicological study of BS showed a high LD50 in rats (>2 g/kg) [149]. A subsequent study showed that BS had no effect on the reproductive system [150]. It has also been shown that high BS exposure is related to alteration of the G5/8 transporters of the hepatic and intestinal ATP-binding cassette and promotes potential risks to the integrity of the blood–brain barrier in diabetic rats [151].

Several in vitro and in vivo studies have shown that CGA can protect cells against toxicities induced by chemicals of different classes (e.g., fungal/bacterial toxins, pharmaceuticals, and metals) by controlling the overproduction of nitric oxide and ROS and suppressed pro-apoptotic signaling [152].

Cos et al. (2001) [153] determined the kaempferol genotoxicity and attributed it mainly to its prooxidative activity. For their part, Ren et al. (2019) generated an antioxidant selectivity index (ASI), which is the maximum non-toxic dose divided by the IC50 value, which is used to assess the toxicity of flavonoids. If the ASI of the flavonoids is >100, they can be considered safe with a good antioxidant activity profile; however, further research is required to standardize it.

Spagnuolo et al., 2012 [154] carried out a study of quercetin toxicity in male F344/N rats fed 2 g per kg body weight per day of quercetin. The results showed severe chronic nephropathy, hyperplasia, and kidney tubular epithelium neoplasm after the treatment. In another study, it was reported that maternal intake of quercetin during pregnancy could increase the risk of mixed lineage leukemia (MLL) gene rearrangements, which is common in childhood leukemia, especially in the presence of compromised DNA repair [155]. However, Utesch et al. (2008) [156] demonstrated that orally administered quercetin (up to 2 g/kg body weight/day) did not induce unscheduled DNA synthesis in male or female rat hepatocytes, suggesting that quercetin was not genotoxic. Additionally, in the only phase I clinical trial of quercetin found, renal toxicity was detected without signs of nephritis or obstructive uropathy when a dose of 50 mg/kg (approximately 3.5 g/70 kg) was administered by intravenous infusion at three weeks or at weekly intervals [157].

## 5. *Eryngium* spp. Propagation Strategies

Given the wide variety of uses that different species of the genus *Eryngium* have, it is important to develop strategies to cultivate them, but some limitations should be faced, such as non-synchronized or uniform seed germination, low germination rates, lack of seed availability, and the need to reach higher sowing rates [158]. In this sense, due to the scarce reports found in literature about the propagation of the *Eryngium* species located in Mexico, particularly in the central-western region, some works in other species were reviewed as a starting point for future development in this matter.

Mozumder et al. (2010) [159] achieved an increase of *E. foetidum* germination rate, this species is of great importance in Latin America and other parts of the world. The authors reported that gibberellic acid (GA3, 1000 ppm) and kinetin (50 ppm) were effective to promote germination up to 28.54%. In a complementary work, Mozumder et al. (2017a) [158] evaluated different storage conditions, application of growth regulators, and soaking to increase *E. foetidum* seeds’ germination rate. Seeds kept at low temperature (3–5 °C) had a germination percentage of 18.4% and increased to 32.3% with 12 h of soaking and the addition of growth regulators. Mozumder et al. (2017 b) [160] evaluated seed germination and field performance of *E. foetidum* using soaking, growth regulators, and pesticide (0.2% copper oxychloride + 1000 ppm tetracycline) combinations. The maximum percentage of germination (74.7%) and early germination (12 days) was obtained using growth regulators (GA3 500 ppm and kinetin 50 ppm) with 96 h of soaking. Additionally, Mozumder et al. (2017c) [161] evaluated the growth and biomass production observing that the profitability increased, and the seed production cost decreased; the authors reported the highest values of the following variables: germination percentage, number of seedlings, number of harvestable plants/m^2^, number of leaves per plant, leaf width and biomass, at 30 and 60 days with the application of growth regulators.

On the other hand, Mozumder et al. (2020) [162] used three different levels of shades and two planting methods at two locations to cultivate *E. foetidum*. The maximum gross yield (4944.2 thousand TK/ha) and the net yield (4438.2 thousand TK/ha) were obtained with the shade of nylon netting in broadcast planting, which was better to produce leaves and obtain higher profits. These results coincided with those reported by Kuttan (2008) [163] who cultivated *E. foetidum* at three shade levels and four plant spacings. The maximum yield was obtained with a shade level of 75% (1411.04 g/plot size 120 × 150 cm). From storage studies it was concluded that at room temperature the leaves could be stored for a maximum of 5.2 days with a shade level of 75%, while under cold storage conditions and a shade level of 75% the leaves could be stored for a maximum of 109.65 days without any deterioration. The highest cost-benefit ratio (1.28) was obtained with a shade level of 75% and 15 cm × 15 cm spacing.

One of the few published works reporting the propagation of *Eryngium* species in Mexico is that of Carrera-Quirino and Colohua-Citlahua (2014) [164] who generated a reproduction strategy for *Eryngium proteaeflorum* in a nursery. The authors established recommendations for the collection of germplasm, characteristics of the mother plants, collection strategies to avoid damaging the habitat of the species and allow natural regeneration, as well as for propagation in seedbeds and transplant to planting bags.

Another propagation method reported for *Eryngium* spp. is the vegetative tissue culture (e.g., leaf, stem, or root segments) using culture media such as Murashige and Skoog (MS). The use of basal medium supplemented or not with sucrose, macro and micronutrients has been suggested [165]. Additionally, different explants and growth regulators, such as indole butyric acid (IBA) and indole acetic acid (IAA), have been used in various combinations [166], and the treatment with IBA 500 ppm and 500 ppm IAA using 7 to 10 cm shoots was the best to promote growth and obtain commercial seedlings. On the other hand, Ayuso et al. (2019) [167] reported the use of different growth inducers, sucrose, and salt concentrations for the propagation of *E. viviparum*, achieving a 96% survival rate in plants. In the same way, Martin (2006) [168], Nagananda et al. (2012) [169], and Jena et al. (2020) [170] reported protocols for the fastest and most successful clonal propagation of *E. foetidum* reaching an 85–90% plant survival rate using different explants, doses of inducers, and nitrogen sources.

## 6. Conclusions

The use of plants belonging to the genus *Eryngium* is widely distributed around the world, especially for medicinal and culinary purposes. The species of the genus whose presence and use in phytotherapy have been reported in central-western Mexico, especially in the states of Michoacán and Jalisco, are *E. cymosum*, *E. longifolium*, *E. fluitans* (or *mexicanum*), *E. beecheyanum*, *E. carlinae*, *E. comosum*, *E. heterophyllum*, and *E. nasturtiifolium*. The use of which has been recorded for the treatment of at least 27 ailments, since diverse benefits are attributed to them, such as antibacterial, anticrotalic, antispasmodic, antigonorrheal, anti-inflammatory, antioxidant, antipodagric, antitumor, carminative, digestive, hypoglycemic, hypocholesterolemic, and diuretic, among others; therefore, they are considered of great significance in the local populations’ health care. However, not all of them have been validated by scientific studies of the corresponding biological activities. Studies of biological activities have been reported only for five of them, and some of the metabolites that four of these *Eryngium* spp. contain, have been identified.

*E. carlinae* is the most studied species, and its hypoglycemic and hypolipidemic properties have been confirmed both in vitro and in vivo models. In addition, it has shown antioxidant, antimicrobial, and anti-inflammatory activities. Regarding its phytochemistry, more than 30 different compounds belonging to various groups of metabolites have been identified in *E. carlinae extracts*: hydroxybenzoic acids, hydroxycinnamic acids, phenolic acids, flavonoids, flavanols, flavanones, hydroxybenzaldehydes, phytosterols, saponins, terpenes, and sesquiterpenes. Much less information about the other species is published, and there are fewer toxicity studies. Therefore, more research is required on its phytochemistry and in vitro and in vivo biological activities. It is recommended to carry out in vivo tests with the extracts and metabolites of the *Eryngium* species that have been studied in vitro, and to later carry out clinical trials on inflammatory, infectious, and chronic diseases. An important aspect to investigate is the elucidation of the mechanisms of action associated with the beneficial effects reported. Additionally, the separation, isolation, and identification of the active metabolites or bioactive compounds must be performed since the greatest advances are made only in *E. carlinae*, followed by *E. cymosum* and *E. longifolium*. Having this information will allow the development of effective and safe treatments, drugs, and supplements, in addition to validate and promote the use of this plant resource for medicinal purposes in the communities that possess them. However, toxicity issues need to be addressed.

This literature review did not show any reports on the propagation or cultivation strategies of the *Eryngium* spp. addressed in this review, so it is necessary to start developing them to achieve the sustainable use of this important plant resource since they are obtained mainly by collection, causing populations to decline.

## Figures and Tables

**Table 1:** *Eryngium* species reported in the central-western region of Mexico.

Specie	Distribution
*E. alternatum*	State of Mexico, Jalisco, and Michoacán
*E. beecheyanum*	State of Mexico, Guanajuato, Jalisco, and Michoacán
*E. bonplandii*	State of Mexico, Jalisco, Guanajuato, and Michoacán
*E. carlinae*	State of Mexico, Guanajuato, Jalisco, and Michoacán
*E. cervantesii*	State of Mexico, Jalisco, Guanajuato, and Michoacán
*E. columnare*	State of Mexico, Jalisco, and Michoacán
*E. comosum*	State of Mexico and Michoacán
*E. cymosum*	State of Mexico, Jalisco, and Michoacán
*E. ferrisiae*	Jalisco and Nayarit
*E. fluitans (E. mexicanum)*	State of Mexico and Michoacán
*E. ghiesbreghtii*	State of Mexico, Jalisco, and Michoacán
*E. gracile*	State of Mexico, Jalisco, and Michoacán
*E. haenkei (E. spiculosum)*	State of Mexico, Michoacán, and Guerrero
*E. heterophyllum*	State of Mexico, Jalisco, and Michoacán
*E. jaliscience*	Jalisco
*E. longifolium*	State of Mexico, Jalisco, and Michoacán
*E. mexiae*	State of Mexico, Jalisco, and Michoacán
*E. nasturtiifolium*	State of Mexico, Jalisco, and Michoacán
*E. monocephalum*	Guanajuato and Michoacán
*E. palmeri (E. globosum)*	Jalisco and Nayarit
*E. pectinatum*	State of Mexico and Michoacán
*E. phyteumae*	State of Mexico and Michoacán
*E. proteaeflorum*	State of Mexico and Michoacán
*E. pugae*	Jalisco and Aguascalientes
*E. serratum*	State of Mexico, Guanajuato, and Michoacán
*E. sparganophyllum*	Jalisco and Michoacán
*E. subacaule (E. ranunculoides)*	State of Mexico and Michoacán
*E. yuccifolium*	Michoacán (exotic)

**Table 2:** Location of American and Australian (New World) Species and corresponding sections and subsections [18,19].

Section	Subsection	Specie
Carliniformia	Comosa	*E. carlinae*
		*E. comosum*
		*E. beecheyanum*
Madrensia	Setoso-dentata	*E. heterophyllum*
		*E. fluitans*
Reptantia		*E. nasturtiifolium*
Panniculata		*E. longifolium*
Spinescentia		*E. cymosum*

**Table 3:** Some characteristics on the habitat, biology, and distribution of the *Eryngium* species found in the central-western region of Mexico reported with a medicinal use [6,23].

Specie	Habitat	Altitude (m·asl)	Phenology	Known Distribution
*E. beecheyanum*	grassland, tropical deciduous forest, and oak-pine forest	1600–2550	flowers from April to May and bears fruit from June to January	Chiapas, Chihuahua, Tepic, Sinaloa, Zacatecas, the State of Mexico, Jalisco, Colima, Michoacán, Oaxaca, and Sonora; Central America
*E.carlinae*	prairies, pastures, disturbed oak, pine-oak and coniferous forest	1500–3500	flowers from May to July and bears fruit from August to January	Chihuahua, Federal District, Durango, State of Mexico, Michoacán, Hidalgo, and Oaxaca; Central and South América Guatemala, and Costa Rica
*E. comosum*	plains and grasslands from thorny scrub and oak forest clearings	1900–2300	flowers from June to August and bears fruit from September to November	Federal District, Guerrero, Hidalgo, Michoacán, and Querétaro
*E. cymosum*	slopes and understory of oak forest and coniferous forests	2000–3200	flowers from July to October and bears fruit from October to January	State of Mexico, Guerrero, Hidalgo, and Michoacán
*E. fluitans*	humid and flooded grasslands, plains and clearings within oak, pine-oak forests	2250–2700	flowers from July to August and bears fruit from September to November	Chihuahua, Durango, the State of Mexico, Michoacán, and Morelos
*E. heterophyllum*	grassland and secondary vegetation, tropical deciduous, and oak forests	1800–2000	flowers from July to August and bears fruit from September to October	Louisiana, Texas, and Arizona in the United States of America; in Mexico it is in Chihuahua, Durango, San Luis Potosí, Jalisco, Michoacán, State of Mexico, and Oaxaca
*E. longifolium*	plains and slopes of oak, oak-pine forests, and grasslands	1500–2400	flowers from July to August and bears fruit from September to December	Durango, the State of Mexico, Guerrero, Hidalgo, Jalisco, Michoacán, and Oaxaca
*E. nasturtiifolium*	semi-humid environments on the edges of cultivated and disturbed lands, grasslands, and scrublands	1000–1800	blooms from February to May and bears fruit from May to August	Southern Arizona and Texas in the United States of America; Mexico: Baja California, Sonora, Sinaloa, Tamaulipas, Veracruz, Nuevo León, Nayarit, Jalisco, and Michoacán to Oaxaca; Cuba, and Central America

**Table 5:** Confirmed biological activities in *Eryngium* species distributed in the central-western region of Mexico.

*Eryngium* sp.	Biological Activity Confirmed	Type of Extract	Plant Tissue	Model	Reference
*E. carlinae*	Hypolipidemic	Ethanolic	Plant	In vivo	[31,50]
		Aqueous	Plant	Clinical trial	[32]
		Hexanic	Inflorescence	In vitro and in vivo	[52]
	Hypocholesterolemic	Ethanolic	Plant	In vivo	[31,51]
		Hydroalcoholic	Aerial parts	In vivo	[53]
	Hypoglycemic	Hexanic	Inflorescence	In vivo	[52]
		Hexanic	Inflorescence	I vitro and in vivo	[30]
	Antioxidant	Hydroalcoholic	Aerial parts	In vivo	[53]
		Ethanolic	Plant	In vivo	[51]
		Hexanic	Inflorescence	In vitro and in vivo	[30]
	Diuretic-Renoprotective	Decoction	Plant	In vivo	[55]
	Antimicrobiane (*Pyhytophtora cinnamomi*)	Ethanolic	Aerial parts	In vitro	[59]
	Anticholelithiasis	Hydroalcoholic	Plant	In vitro	[56]
	Antispasmodic	Methanolic	Aerial parts	In vivo	[57]
*E. comosum*	Hypocholesterolemic	Aqueous	Plant	In vivo	[13]
	Antioxidant	Aqueous and ethanolic	Plant	In vitro	[60]
		Ethanolic	Plant	In vitro	[62]
	Antimicrobiane (Equine pathogens)	Ethanolic	Plant	In vitro	[61]
	Cytotoxicity	Ethanolic	Plant	In vitro	[62]
		Aqueous and ethanolic	Plant	In vitro and in vivo	[60]
*E. cymosum*	Hypoglycemic	Aqueous	Aerial parts	In vivo	[8]
	Antihyperglycemic	Infusion	Plant	In vivo	[36]
*E. heterophyllum*	Trypanocide	Methanolic	Aerial parts		[66]
	Hypocholesterolemic	Aqueous	Plant	In vivo	[64]
	Hypocholesterolemic	Decoction	Plant	Clinical trial	[65]
*E. longifolium*	Hypoglycemic	Aqueous and ethanolic	Aerial parts	In vivo	[35]

Plant—plant parts were not specified.

**Table 6:** *Eryngium* spp. distributed in the central-western region of Mexico whose phytochemistry has been analyzed.

Species Analyzed	Plant Tissue Analyzed; Type of Extract	Analysis Technique	Group of Metabolites Detected	Compounds Detected	Quantification	Reference	Other *Eryngium* Species Containing
*E. carlinae*	Aerial parts (stem, leaves, and inflorescences); methanolic	Phytochemical screening	Tannins Saponins Flavonoids	NA	Detection only	[11]	*E. pyramidale*, *E. foetidum*, and *E. creticum* [74,88,89]
*E. carlinae*	Aerial parts; aqueous (infusion)	UV-Vis	Total Phenol (Folin–Ciocalteu method) Total flavonoids Total terpenoids	NA	0.0038 mg GAE/mL 3.3032 mg QE/mL 0.0424 mg LE/mL	[12]	*E. creticum* and *E. maritimum* [74,90]
*E. carlinae*	Aerial parts; aqueous (infusions at 1 and 2% *m*/*v*; decoctions at 5 and 10 min)	UV-Vis	Total Phenol (Folin–Ciocalteu method) Total flavonoids	NA	≥2.5 mg GAE/mL ≤0.2 mg QE/mL	[32]	*E. pyramidale*, *E. creticum*, and *E. foetidum* [74,88]
		HPLC-DAD-MS	Hydroxybenzoic acids	Gallic acid 4-Hydroxybenzoic acid Protocateuic acid	1.3–2.6 µg/mL 212–332 µg/mL 105–210 mg/mL	[32]	*E. planum*, *E. campestre*, *E. maritumum*, *E. alpinum*, *E. foetidum*, *E. bornmuelleri*, and *E. caucasicum* [75,91,92,93,94,95,96]
			Hydroxycinnamic acids	Chlorogenic acid Rosmarinic acid Caffeic acid Ellagic acid P-coumaric acid Ferulic acid Synapic acid	400–700 μg/mL 40–58 μg/mL 12–21 μg/mL 78–116 μg/mL 19–32 μg/mL 10–16.5 μg/mL 2–3.5 μg/mL	[32]	
			Flavanols	Galocatequin gallate Catechin Epicatechin Epigallocatechin gallate	600–1600 μg/mL 68–97 μg/mL 11–16.5 μg/mL 6–10 μg/mL	[32]	
			Flavanones	Quercetin Rutin Kaempferol	89–179 μg/mL 9–156 μg/mL 9–15 μg/mL	[32]	
			Hydroxybenzaldehydes	Eriocitrin Hesperidin Naringenin Vanillin	0.2–0.5 μg/mL 0.4–0.6 μg/mL 0.2–04 μg/mL 4.7–8.4 μg/mL	[32]	
*E. carlinae*	Aerial parts; aqueous (infusion)	HPLC-DAD	Phenolic acids Flavonoids Phytosterols Saponins	Ellagic acid Caffeic acid Protocateuic acid P-hydroxybenzoic acid Rutin Catechin Epicatechin α-7-stigmasterol β-sitosterol β-campesterol Stigmastanol Campsteryl β-d-glucopyranoside Sitosteryl β-d-glucopyranoside	38.3 mA 20.3 mA 11.9 mA 9.8 mA 14.1 mA 12.1 mA 11.7 mA 18.7 mA 11.1 mA 8.7 mA 8.4 mA 28.9 mA 20.1 mA	[55]	
*E. carlinae*	Inflorescence; hexanic	GC-MS	Terpenes Sesquiterpenes	(Z) β-farnesene β-pinene Calamenene α-farnesene	38.79% 17.53% 13.3% 10.38%	[30]	*E. glaciale*, *E. amethystinum*, *E. campestre*, *E. thorifolium*, *E.* *Creticum*, *E. pristis*, *E. maritimum*, *E. alpinum*, and *E. rosulatum* [97,98,99,100,101,102]
*E. carlinae*	Aerial parts; ethanolic	GC-MS	Sesquiterpenes Fatty acids	α-selinene β-selinene Palmitic acid Stearic acid Humulene Stigmasterol Elemol Elemene α-cedrene	17.54% 26.04% 14.43% 14.53% ≤5%	[31]	
*E. carlinae*	Aerial parts; hydroalcoholic (EtOH-H20, 7:3 *v*/*v*)	GC-MS	Saccharides Polyols	Hexa-*O*-acetyl-d-mannitol, acetylated derivatives thereof	Detection only	[53]	*E. dichotomum* [103]
*E. carlinae*	Leaves and stems; ethanolic	GC-MS	Saccharides Polyols Organic acids	D-(−)-fructofuranose D-(−)-fructopyranose D-(−)-tagatofuranosa 1,5-anhydro-d-sorbitol Cinnamic acid	Detection only	[87]	*E. dichotomum* and *E. bourgatii* [103,104]
*E. carlinae*	Aerial parts (leaves + inflorescences); ethanolic	GC-MS	Terpenoids	Borneol α-pinene Myrcene Caryophyllene β-pinene	367 mg/L 278 mg/L 256 mg/mL 225 mg/mL 120 mg/mL	[59]	*E. alpinum*, *E. amethystinum*, and *E. bungei* [105,106]
*E. carlinae*	Aerial parts; ethanolic	Phytochemical screening	Triterpenoids Sterols Tannins Coumarins Carboxyls Flavonoids Carbohydrates	NA	Detection only	[58]	*E. pyramidale*, *E. foetidum*, and *E. creticum* [74,88,89]
*E. comosum*	Aerial parts; aqueous (decoction)	Phytochemical screening	Alkaloids Flavonoids (flavones and xanthones) Triterpenoid saponins Reducing sugars Tannins derived from catechol Phenolic compounds Benzoquinones	NA	NA	[13]	
*E. comosum*	Aerial parts; aqueous, and hidroethanolic 50% and 70%	UV-Vis	Total Phenol Total Phenol (Folin–Ciocalteu method) Total saponins	NA	8.0–13.3 mg/mg 0.69–4.33 mg GAE/mg 0–29.33 mg/g DW	[62]	*E. creticum* and *E. maritimum* [74,90]
*E. comosum*	Aerial parts; ethanolic	UV-Vis	Total Phenol Total Phenol (Folin–Ciocalteu method) Total saponins	NA	22.1 mg/mg 4.33 mg GAE/g dry weight (DW) 62.2 mg/g DW	[61]	
*E. longifolium*	Aerial parts; aqueous (infusion)	HPLC	Phenolic acids Isoflavones Glycosylated flavonoids	Caffeic, chlorogenic acid, and rosmarinic acid	Detection only	[35]	*E. planum*, *E. campestre*, *E. maritumum*, *E. alpinum*, *E. foetidum*, *E. bornmuelleri*, and *E. caucasicum* [43,75,91,92,93,94,95,96,107]
*E. cymosum*	Aerial parts; aqueous (infusion)	HPLC	Phenolic acids	Caffeic, chlorogenic acid, and rosmarinic acid	Detection only	[8]	
*E. cymosum*	Aerial parts; aqueous (infusion), ethanolic, and organic (butanol)	HPLC, NMR	Phenolic acids Flavonoids	Chlorogenic and rosmarinic acid (EA) Caffeic and protocateuic acid Kaempferol-3-*O*-(2,6-di-*O*-trans-p-coumaryl)-β-d-glucopyranoside	Detection only	[40]	

NA, not applicable; UV-Vis, ultraviolet visible spectrophotometry; HPLC-DAD-MS, high-performance liquid chromatography with a diode array detector coupled to mass spectrometry; HPLC-DAD, high-performance liquid chromatography with a diode array detector; GC-MS, gas chromatography coupled to mass spectrometry; NMR, nuclear magnetic resonance.

## Data Availability

Not applicable.
